# Ischemic Stroke in a Patient With Polyneuropathy, Organomegaly, Endocrinopathy, Monoclonal Protein, and Skin Changes Syndrome Treated With Lenalidomide

**DOI:** 10.7759/cureus.9346

**Published:** 2020-07-22

**Authors:** Soujanya Sodavarapu, Arshian Mahajan

**Affiliations:** 1 Internal Medicine, San Joaquin General Hospital, French Camp, USA; 2 Family Medicine, San Joaquin General Hsopital, French Camp, USA

**Keywords:** poems syndrome, lenalidomide

## Abstract

Polyneuropathy, organomegaly, endocrinopathy, monoclonal protein, and skin changes (POEMS) syndrome is an uncommon multisystemic disease associated with plasma cell dyscrasia. Due to the disease's rarity and an even rarer presentation of stroke in afflicted patients, a direct association between POEMS syndrome and stroke remains ambiguous. Thrombocytosis, hyperfibrinogenemia, and increased levels of inflammatory cytokines occur in this disease, which can predispose patients to thromboembolic events. Immunomodulators can also enhance thrombosis, the chances of which increase when they are combined with dexamethasone. We present a case of a 28-year-old patient with an ischemic stroke, which may have been triggered by the combination of POEMS syndrome-associated vasculitis and the thrombogenic nature of lenalidomide-dexamethasone therapy.

## Introduction

Polyneuropathy, organomegaly, endocrinopathy, monoclonal protein, and skin changes (POEMS) syndrome is an uncommon multisystemic disease associated with plasma cell dyscrasia [1]. Thrombocytosis, hyperfibrinogenemia, and increased levels of inflammatory cytokines occur in this disease, and hence the patient may be susceptible to thromboembolic events. However, its association with cerebrovascular events is still being evaluated. Lenalidomide, in combination with dexamethasone, is an effective treatment for POEMS syndrome but poses a high risk of venous thromboembolism [2]. We present a case of a 28-year-old male with ischemic stroke who was diagnosed with POEMS syndrome and was treated with lenalidomide-dexamethasone combination therapy.

## Case presentation

A 28-year-old male with a history of hypertension and aortic stenosis presented with paresthesia of the left foot, stiffness in the lower back and neck, and weight loss of 15 pounds for the past few months. His vitals were stable at presentation. On physical examination, the cranial nerves were intact, while strength and sensation in extremities were normal, except for bilateral foot drop and decreased lower extremity tone. X-rays of the lumbar spine showed sclerosis of the L4-L5 vertebral bodies and small sclerotic densities in the left iliac bone. An MRI showed a large sclerotic lesion at L5 and small sclerotic lesions in the bilateral iliac. A CT scan of the torso showed prominent bilateral axillary and mediastinal lymph nodes, hepatomegaly, splenomegaly, retroperitoneal and iliac lymph node enlargement, and widespread sclerotic foci in the bones. A subsequent ultrasound-guided left inguinal biopsy showed atypical lymphoid proliferation. Moreover, a CT-guided retroperitoneal lymph node biopsy showed mild kappa‑predominant plasmacytosis, with faint monoclonal lambda chains on protein electrophoresis. A lymph node biopsy showed an abundance of plasma cells that were polytypic for kappa and lambda. This was in line with high kappa (56.3 mg/L) and lambda (31.5 mg/L) hematological test values, with a ratio of 1:79. Vascular endothelial growth factor (VEGF) levels were elevated at 1,156 pg/mL, while electromyography studies were consistent with severe axonal loss and demyelination. The patient was subsequently diagnosed with POEMS syndrome.

**Figure 1:**
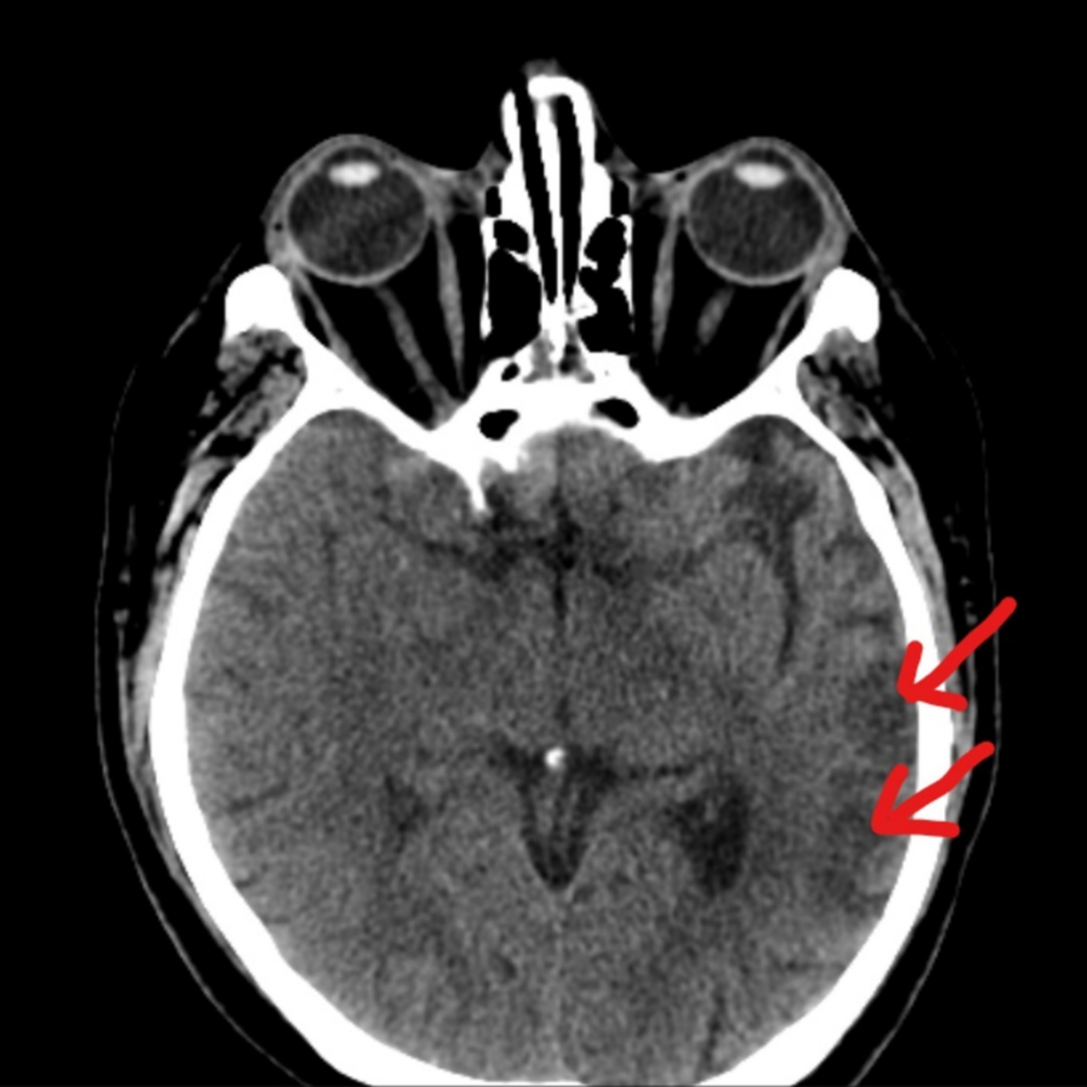
CT of the brain The image shows an acute non-hemorrhagic infarct in the left temporal cortex (arrows) CT: computed tomography

The patient was treated with radiotherapy and lenalidomide with dexamethasone. VEGF levels improved to normal (231 pg/mL). However, he developed a headache and expressive aphasia after three months on lenalidomide. A CT brain revealed an acute non-hemorrhagic infarct in the left temporal cortex (Figure 1), while magnetic resonance angiography showed no signs of atherosclerosis in the cranial vessels. His stroke was presumed to be due to the thrombogenic effect of POEMS syndrome with superimposed thrombogenesis from lenalidomide, which was subsequently stopped while he received aspirin 81 mg and atorvastatin 40 mg daily. He was started on carfilzomib and dexamethasone but did not experience clinical improvement. He is being considered for melphalan and autologous stem cell transplantation.

## Discussion

POEMS syndrome is a rare, life-threatening, and disabling plasma cell disorder where the acronym stands for polyneuropathy, organomegaly, endocrinopathy, monoclonal protein, and skin changes [1]. Though the pathophysiology underlying the disorder remains unknown, it is postulated to be due to various proinflammatory growth factors and cytokines that are thought to stimulate VEGF expression, which likely plays a vital role in the pathogenesis of the disease [2]. Its presentation varies from person to person and, due to its diverse clinical manifestations, especially when presented to a primary care provider, can have a longer lead time for diagnosis ranging from 13-18 months, and could be even misdiagnosed as other disorders such as chronic inflammatory demyelinating polyneuropathy, amyloidosis, and multiple myeloma [2]. Diagnosis of POEMS syndrome requires the fulfillment of two mandatory criteria, one or more major criteria, and one or more minor criteria (Table 1) [3].

**Table 1:** Diagnostic criteria for POEMS syndrome POEMS syndrome: polyneuropathy, organomegaly, endocrinopathy, monoclonal protein, and skin changes syndrome

Criteria, symptoms, and diagnosis	Description
Mandatory criteria	1. Polyneuropathy (typically demyelinating). 2. Monoclonal plasma cell-proliferation disorder (almost always λ)
Major criteria	1. Sclerotic bone lesions. 2. Castleman disease. 3. Vascular endothelial growth factor elevation
Minor criteria	1. Organomegaly (splenomegaly, hepatomegaly, or lymphadenopathy). 2. Extravascular volume overload (edema, pleural effusion, or ascites). 3. Endocrinopathy (adrenal, thyroid, pituitary, gonadal, parathyroid, pancreatic). 4. Skin changes (hyperpigmentation, hypertrichosis, glomeruloid hemangiomata, plethora, acrocyanosis, flushing, and white nails). 5. Papilledema. 6. Thrombocytosis/polycythemia
Other symptoms and signs	Clubbing, weight loss, hyperhidrosis, pulmonary hypertension/restrictive lung disease, thrombotic diatheses, diarrhea, low vitamin B12 values
Diagnosis of POEMS syndrome	Requires the fulfillment of two mandatory criteria + ≥1 major + ≥1 minor criteria

Due to the disease's rarity and an even rarer presentation of stroke in afflicted patients, a direct association between POEMS syndrome and stroke remains ambiguous. Dupont et al. have described a case series of 208 patients, of which 19 patients (9.2%) developed cerebral infarction with a median age of 53 years (range: 36-77 years) [4]. Acute vascular angiopathy and macroangiopathy from the increased inflammatory growth factors and VEGF are assumed to cause vasculitis and vascular anomaly in these patients [5,6]. High levels of proinflammatory cytokines with hyperfibrinogenemia, along with hyperviscosity and polyglobuminemia, can augment the hypercoagulable state [7]. These mechanisms can cause cerebrovascular accidents in patients with POEMS syndrome. More cases have been reported regarding limb and coronary artery involvement, but cerebrovascular involvement has been rarely reported [6]. Cerebral infarction has been noted in only two cases who were diagnosed with POEMS syndrome and were on lenalidomide-dexamethasone combination therapy [8].

Treatment for POEMS syndrome ranges from radiation, corticosteroids, chemotherapy agents like alkylating agents (melphalan), and immunomodulators (thalidomide and lenalidomide) to targeted therapy with protease inhibitors (bortezomib) and autologous stem cell transplantation. The use of thalidomide, lenalidomide, and bortezomib have shown promising results with significant clinical improvements [9].

Immunomodulators (e.g., thalidomide, lenalidomide) have been associated with thromboembolism. Immunomodulators can increase VEGF, increase the release of cytokines and cause activated protein C resistance, downregulate thromboplastin, and regulate cyclooxygenase-2 (COX-2) prothrombotic factor. They have been shown to increase the levels of von Willebrand factor and factor VIII, which cause venous and arterial thrombosis [10]. When they are co-administered with corticosteroids, it further increases the risk of thromboembolism [9]. In two phase-3 randomized control trials involving multiple myeloma patients, a combination of lenalidomide with dexamethasone was associated with 3.4% of cerebrovascular events compared to 1.7% with dexamethasone alone [11].

In her 2019 update on the various aspects of POEMS syndrome, Angela Dispenzieri did mention prophylactic use of aspirin, as well as the use of low-molecular-weight heparin and warfarin in patients with POEMS syndrome, weighing against their risk factors; however, there have been no studies documenting the prophylactic use of these drugs in this disease so far [3].

## Conclusions

The ischemic event in our patient may have been caused by the combination of POEMS syndrome-associated vasculitis and the thrombogenic nature of lenalidomide-dexamethasone therapy. Further studies are needed to confirm the correlation between vasculitis-induced stroke and lenalidomide-dexamethasone therapy in POEMS syndrome patients.
